# Annotations

**Published:** 1899-04-08

**Authors:** 


### Annotations.

### Civilisation and Typhoid Fever.

Typhoid fever still remains the centre of interest in Philadelphia, where during the eleven weeks intervening between the beginning of January and March 18th there have been no less than 4,126 cases with 424 deaths. It is clear that all over the Union the prevalence of typhoid fever is becoming a very serious matter, and that the lessons which have been learned by painful experience in older countries will have to be taken to heart also in America. In the earlier days the States revelling in the luxury of an unlimited territory could safely indulge in a certain laxity in sanitary arrangements, and especially in regard to water supply. Townships and cities stood far apart, and not only did large tracts of virgin land often intervene between them, but the towns themselves were in many cases planned on a much more liberal scale and made to cover a much larger area than had been usual in Europe. Gradually, however, the States have become, to a large extent, settled up; as in older countries, the rivers have become great drains, and the self-purifying processes by the aid of which all great rivers are able to some extent to sweeten themselves have become insufficient in view of the increasing mass of sewage finding its way into them, and the steadily lessening distance between the large centres of population on their banks. Moreover, another influence, and one which has probably been still more effectual for evil has been at work, namely, the enormous suburban growth with which all great cities have surrounded themselves. This is not peculiar to the States. Everywhere cities have become surrounded, and it is to be feared that their sources of water supply have often become fouled, by a vast suburban zone of villas, villages, and small towns; and where, as is the case with many of the cities of the United States, the rivers on which they stand are largely used both for purposes of traffic and as sources of water supply, the result has been disastrous.

North America is wonderfully supplied with rivers, and the inhabitants of that country have made full use of them. They have travelled on them, they have drained into them, and they have drunk out of them, and for a long time the bounteous provision of space and distance which nature gave them prevented the evils being so great as they have since become. But no animal can go on for long fouling its own nest without suffering the consequences—man, least of all—and there can be no doubt that if America be wise she will accept the lesson which this recent outbreak in Philadelphia serves to drive home so forcibly, namely, that her cities must either provide filtered water for their citizens, or must dedicate to water-gathering purposes large areas of such superfluous lands as may be within reach. In some places the one course, in some the other, will be the most feasible; but in any case a great civilised nation cannot afford to neglect one of the first duties of civilisation, namely, the protection of its water supplies. How expensive an affair this becomes when it is put off too long London will probably soon see, and the Nemesis of civilisation is just the same on one side of the Atlantic as on the other. Nor is this a matter entirely of cities. There, as here, those parts of the country which have a smaller proportion of town population have a relatively larger proportion of typhoid mortality. With the settling up of a country, with the fouling of its water sources, comes punishment in the form of typhoid fever. Dr. William Osler, speaking on this subject before the Medical Society of the State of New York recently, said: "Last autumn this nation in the moment of victory had a rude awakening, a sudden conviction, a hard lesson. A voice like that heard in Ramah went up throughout the land. . . . Rachels weeping for their lads cut off by a cruel disease. . . . To us these autumnal dirges rang no new tune. . . . . Year by year we had listened to Rachels of this land weeping for their fair sons and fairer daughters, not killed by any pestilence that walked in darkness, but by a preventable sickness. . . . Throughout the length and breadth of the land typhoid fever prevails so extensively in township and county, in village and city, that any large body of men aggregated together is almost certain to become infected." Unfortunately, this is true of all countries. Directly precautions are relaxed typhoid becomes a serious factor in the death-rate, a fact of which we have had many warnings in recent years.

### Who is to Certify?

A question of considerable ethical interest is raised by an editorial comment in the *British Medical Journal* of March 25th. A correspondent writing on the question of hospitals and certificates under the Workmen's Compensation Act said that "the majority of the claimants under the Act naturally enough pass right from the accident into the hospitals, and it is the house-surgeon, and the house-surgeon only, who can give the certificate and information required." On this apparently very simple proposition the editor commented as follows: "Our correspondent is mistaken in thinking that 'the house-surgeon, and the house-surgeon only, can give the certificate and the information required.' In proof of this we can assure him that in larger centres than Birkenhead, and in larger hospitals than the Birkenhead Hospital such certifi-

ANNOTATIONS—*continued.*

cates are not given by the resident staff, and the fees are not diverted from the pockets of the medical practitioners of the town and district." This reads like a snub! However, in the next number of the journal there appeared a letter from Mr. Hamilton A. Ballance, Assistant Surgeon to the Norfolk and Norwich Hospital, in which he says what we think can hardly be disputed, namely: "It is undeniable that the house-surgeon, having attended the patient at his accident and during convalescence, is the best person to fill in an accurate certificate, and that without consultation with him a certificate signed by a doctor outside the hospital would possess no claim to scientific accuracy." This exactly expresses the position. What is the use or the authority of a "certificate" which certifies to facts which are not within the cognisance of the man that signs it? It is just such documents that have brought all the discredit on medical certificates, and we think that the readers of the *British Medical Journal* may very fairly ask their editor to define more clearly than he has done how the medical men of the town and district are to ascertain of their own knowledge the facts regarding which the certificate is required in the particular case on which he comments, namely, where the claimants "pass right from the accident into the hospital."

### Death from Drowning.

The terrible catastrophe off the Casquets, which has marred the brightness of an otherwise perfect holiday season, has raised many questions in regard to death by drowning. It is generally considered that drowning causes death either by syncope or asphyxia, and with certain reservations this no doubt is true. What, then, about those who do not die from syncope, and who by the aid of properly applied life-belts are prevented from at once dying of asphyxia? The answer is that they mostly die from cold, although probably the final process in many cases is one of drowning, the fading consciousness permitting the mouth to get under water. The first effect of exposure to cold is to contract the blood-vessels of the skin. This is the first line of defence, the inner parts being thus protected by a layer of skin and fat, in which the circulation is reduced to the lowest possible limits. This, however, is not enough; and, as a second line of defence, the loss of heat through the skin is met to some extent by a great burning up of tissue, as in a fever; and, as in a fever, the waste products of this increased and abnormal metabolism may set up convulsive attacks, or delirium and hallucinations, leading not infrequently to suicide, and ultimately coma. Meanwhile the gradual cooling of the blood lessens the activity of those processes on which life depends, and from this cause also a condition of unconsciousness is induced which quickly ends in death. It is impossible to say how long life may be supported under such circumstances. Often, no doubt, it is shortened by the violent efforts made at first, and often also by the fact that in the first plunge, during which of course the life-belt is no protection against complete immersion, much water is drawn into the lungs, so that the sufferer, being half-drowned to begin with, has but small chance of making a good fight for life; but even if a man were placed quietly in the water he would not live for many hours—how many would depend on his heart and his condition. But the large number of deaths that occurred in the boat which was picked up off Cherbourg—deaths which resulted from nothing but exposure—show plainly how quickly loss of heat may kill, even though the temperature to which the body is exposed may not be very low.

### Speaking-Tubes.

The old story of infection by means of speaking-tubes has cropped up again, as we hope it will continue to do until some improvement is made in this means of communication. Placing the mouth to a tube into which fifty other people have been breathing, and sometimes blowing with all their might, is a disgusting performance worthy only of such a stage of civilisation as permits of feeding off a common plate, and both the danger and the dirtiness of the process are increased by the fact that the tubes are usually entirely unventilated, being plugged at both ends. Now, although in some instances, as where the tube communicates with a private office, this plugging is done for the sake of preventing conversation being heard when not intended, its main use is to enable attention to be drawn by aid of the horrible cacophony of the speaking-tube whistle, and it is perfectly clear that the very simple addition of an electric bell would in most cases do away at once with all necessity for the plug, and would thus allow the tube to be continuously ventilated. Where the tube must be closed at one end it clearly would be by no means impossible to make it, when not in use, plug on to a tube communicating with the open air, or with a chimney, and thus ensure a continuous draught throughout its entire length. The filthy character of many of the speaking tubes through which the dwellers in "Flats" communicate with the porters and the tradesmen calling for orders has long been recognised, and we believe that in the newer blocks of "Mansions" and in all modern hospitals telephones are much used in place of the older arrangement. In fact, when the distance to be traversed is considerable the economy of a wire instead of a tube is so great as to go far to repay the extra cost of the telephone terminal.

### Tuberculosis Among Sailors.

The conditions of life among sailors are such as greatly to encourage the dissemination of tuberculosis unless the most careful measures be taken to prevent the fouling of their quarters, and the maintenance of such discipline as shall do this seems not always to be found an easy task. From a report recently published on the subject it appears that phthisis is far too common in the French navy. "Considering the care exercised in selection, and all the other precautions adopted, the result ought to be a complete absence of all consumptives on board our vessels," says Dr. Vincent in his report, "but unhappily this is only true for a very brief period after each fresh commissioning." It seems to be the dirty habit of expectorating which is at the bottom of the mischief. Spittoons are duly provided and the most stringent notices are posted up conspicuously, but in spite of the most absolute prohibition the men persist in spitting nearly everywhere.

